# Leprosy and the Charity of the Church

**Published:** 1897-08

**Authors:** 


LEPROSY AND THE CHARITY OF THE CHURCH. By Rev. L. W. Mulhane. Chicago and New York: D. H. McBride & Co.

This little volume of 153 pages gives the record of the Catholic Church in ameliorating the condition of lepers, and surely it is a grand one.

The particular record which this book commemorates is that of the noble Father Joseph Damien de Veuster, usually called simply Father Damien, or the "Hero of Molokai." He was born at Tremeloo, Louvain, Belgium, January 3d, 1840.

A request having come from Honolulu, in the Sandwich Islands, for a priest to minister to the lepers in the leper colony of Molokai, near Honolulu, this heroic young man offered himself for the great self-abnegating work, well knowing that his own fate would be leprosy if he undertook it. Being accepted by the Bishop, and having previously been duly ordained priest, he started on his mission, located himself among the lepers, served them in every possible capacity, even with his own hands building a little church and substantial huts to shelter them, dressing their sores, and giving them spiritual consolation, he continued this self-sacrificing work for sixteen years. When he began "he had no church, no house, no purse." He was obliged to sleep for many weeks under a screw-pine tree. Under this same tree he now sleeps the sleep of death. After he had been a minister to these wretched people for eleven years he himself took the disease and lived with it ravaging his system for five years, all the time performing his usual duties until at last he was no longer able to be about, and he, too, became a charge upon another one to minister to him. That other was Joseph Dutton, an American, who had been a soldier in the United States army during the war of the rebellion, had risen to the rank of major, and after the war had met Father Damien and had become his friend and helper in the work.

Father Damien died April 15th, 1889, and was buried amid the scene of his labors.

In reading this touching account of this heroic man with his incredible self-sacrifice one is reminded of the history of those intrepid Jesuit missionaries who came to Quebec in the early part of the last century to minister to the Canadian Indians distributed along the valley of the St. Lawrence River, were captured by the ferocious Iroquois of New York, dragged almost bare-footed over the snow for hundreds of miles, brought down to the vicinity of what is now New York City, tortured with fire and every other damnable device that the savage mind could suggest, escaping through the help of the Dutch burghers of New York, returning to France only to set out again to the scene of their former labors, sufferings, and perils. This steadfastness of purpose and wonderful self-sacrifice and endurance for the sake of benefiting their fellows and converting them is a characteristic of the priests of the Catholic Church, and history is full of their deeds of heroism. Father Damien adds another to the list.

